# Dietary Pea Fibre Improves Obesity, Intestinal Barrier, Reproductive Performance, Offspring Health of Parent Mice Deprived of Dietary Fibre

**DOI:** 10.3390/ani15050655

**Published:** 2025-02-24

**Authors:** Siqi Tian, Mi Zhang, Yanhong Chen, Hanqing Sun, Qingqing Li, Yajin Yang, Aiwei Guo

**Affiliations:** College of Biological Science and Food Engineering, Southwest Forestry University, No. 300 Bailongsi, Panlong District, Kunming 650224, China; siqitian623@163.com (S.T.); zm879967@163.com (M.Z.); chenyanhong@swfu.edu.cn (Y.C.); sunhq08@swfu.edu.cn (H.S.); doublelqq@163.com (Q.L.)

**Keywords:** pea dietary fibre, gut microbiota, parent, offspring, obesity

## Abstract

As a potential high-quality protein food, peas are enriched in protein and fibre. This study investigated the judicious utilisation of pea fibre and the impact of maternal diet on offspring health in mice. Thirty-six eight-week-old, female, healthy C57BL/6J mice were divided into three groups at random (n = 12 per group): deprived fibre diet (DFD), 5% pea fibre diet (LFD), and 10% pea fibre diet (HFD). After weaning, the offspring mice were fed the same diet as their parents; the respective corresponding groups were DFDO, LFDO, and HFDO. Fibre-deprived mice exhibited decreased average litter size, diminished reproductive performance, increased body weight, and intestinal barrier damage. Mice fed pea fibre showed increased litter size, improved fertility rate of parental mice, regulated body weight, and maintained a normal intestinal barrier morphology without inflammatory cell infiltration. Furthermore, 16S rRNA analysis revealed that pea fibre enhanced diversity and richness of the intestinal microbiota and altered microbial composition. Notably, changes in *Lactobacillus* and *Parabacteroides* in fibre-deprived mice suggest that pea fibre might be a potentially beneficial option for neuropsychiatric diseases. In conclusion, supplementing the diet of maternal mice with pea fibre can mitigate the aforementioned issues in their offspring. This study emphasised the crucial role of maternal fibre consumption in increasing litter size, promoting gut health in offspring, and reducing susceptibility to obesity.

## Simple Summary

As a primary dietary fibre source in human nutrition, pea fibre has demonstrated health benefits through the regulation of glucolipid metabolism. It has also been shown to significantly enhance intestinal peristaltic function. However, current evidence regarding the effects of pea fibre on colonic microbiota in animal models remains limited. This study aimed to investigate the impact of pea fibre supplementation on C57BL/6J mice, specifically evaluating growth performance, reproductive outcomes, and colonic microbiota composition in both maternal mice and their offspring. The results revealed that a 10% pea fibre diet improved growth parameters in both generations, attenuated intestinal and hepatic tissue damage, and increased the abundance and diversity of gut microbiota in maternal and offspring mice.

## 1. Introduction

Changes in lifestyle and dietary consumption in developed and developing countries have highlighted the importance of dietary fibre (DF). The widely accepted definition of DF refers to all polysaccharides and lignin that cannot be digested by endogenous human digestive enzymes. DF consists of two parts according to solubility, respectively, soluble DF (SDF) and insoluble DF (IDF). SDF primarily include pectin, tree gum, and some hemicellulose [1]. SDF has a high water retention capacity and viscosity, and thus can delay the emptying rate of the gastrointestinal tract. In addition, SDF produce short-chain fatty acids (SCFAs) after fermentation by intestinal microorganisms. IDF primarily includes cellulose, hemicellulose, lignin, and chitin. IDF can increase the transportation rate and water retention capacity of the intestinal contents, shorten the fermentation time of indigestible foods in the colon, and increase faecal volume [2]. Desai et al. [3] reported that long-term or indirect deprivation of DF in the diet of mice resulted in the use of host-secreted mucin as a nutrient source by the gut microbiota, which caused thinning of the intestinal mucus layer and damage to the intestinal barrier. Infection with *Citrobacter rodentium* led to entry of the bacteria into the intestinal epithelium of mice deprived of DF, which in turn led to fatal colitis. These results suggest that the gut microbiota caused by insufficient DF degrades the colonic mucosal barrier and increases pathogen sensitivity. Mice rich in DF have complete barrier function and reduced susceptibility to intestinal pathogens, indicating that DF can protect intestinal health. The gut microbiota colonises after birth, when the newborn comes into contact with maternal and environmental microbes [4]. The effects of maternal microbes on offspring gut microbiota have been studied. The results indicate that a maternal high-fat diet can cause intestinal microbiota disorders and metabolic disorder in offspring, leading to the programmed development of various diseases, and such negative effects may persist into offspring adulthood [5,6]. The proportion of Firmicutes in mice fed a high-fat diet was higher, the proportion of beneficial microbiota was lower, and the changes in intestinal microbes among offspring were similar to those in maternal mice [6].

The pea plant (*Pisum sativum* L.) is a leguminous plant that is rich in protein, starch, minerals, and DF. Pea plants have high nutritional value. Its IDF is approximately 10% to 15% and its SDF is approximately 2–9% [7]. Pea fibre has a balanced proportion of SDF and IDF, and a good adsorption capacity for cholesterol and glucose. It can regulate the glucose response, lipid metabolism, and intestinal motility [8,9,10]. Adding high concentrations of yellow pea flour can decrease insulin and glucose levels in golden Syrian hamsters [11]. Using methods that include quantitative proteomics and macroproteomics, targeted screening was conducted on 34 types of DF. The analysis indicated that feeding mice pea fibre selectively affected Bacteroides, leading to a significant expansion of *Bacteroides thetaiotaomicron*. This finding indicates that pea fibre is a potential nutritional source for multiple species of gut microbiota and microbiota-accessible carbohydrates, which play important roles in shaping the gut microbiota ecosystem [12]. In addition, studies have found that a low-DF or DF-deprived diet increases the likelihood of chronic diseases, such as diarrhoea in the body [13]. A recent study demonstrated that by-products of peas alter glucose metabolism in humans [8], which may have relevance elsewhere [14].

Previous studies focused more on the effects of processing technology and different genetic varieties of peas on health. There is limited research on the effects of dietary pea fibre on the health of parent mice and offspring. Whether dietary pea fibre supplementation to the diet can improve health of mice and their offspring is unclear. This study explored the rational use of DF and the impact of parental DF deprivation on offspring health, enriching maternal DF nutrition.

## 2. Materials and Methods

### 2.1. Animals and Diets

**Figure 1:**
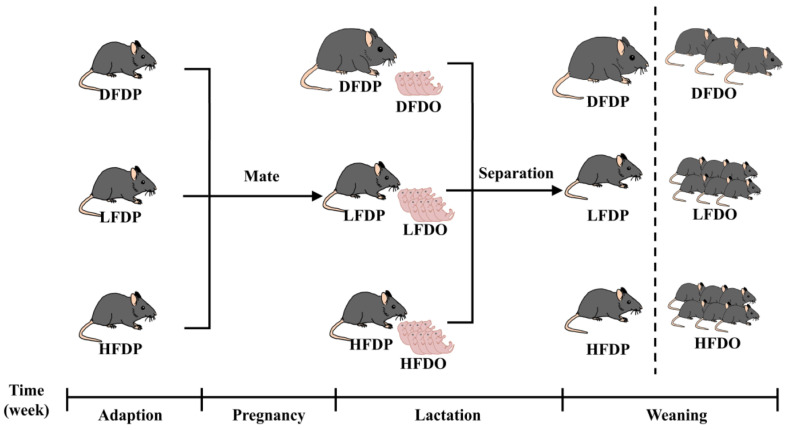
Experiment design. DFDP: deprived-fibre-diet parent mice; LFDP: low-fibre-diet parent mice; HFDP: high-fibre-diet parent mice; DFDO: deprived-fibre-diet offspring mice; LFDO: low-fibre-diet offspring mice; HFDO: high-fibre-diet offspring mice.

Thirty-six 8-weeks-old female C57BL/6J mice with (24.86 ± 0.15) g were selected for study. Trial procedures were approved by the Academic Committee of Southwest Forestry University. Female breeder mice were assigned into three groups at random: deprived fibre diet (DFDP), 5% pea fibre (low-fibre) diet (LFDP), and 10% pea fibre (high-fibre) diet (HFDP), with three replicates and four mice per replicate. After weaning, the offspring mice were fed the same diet as their parents; their respective groups were DFDO, LFDO, and HFDO (Figure 1). Diets were formulated according to the National Research Council recommendations [15] (Table 1). The experimental diet was fed from the beginning to the end of the trial. The maternal mice were fed for a total of 20 weeks, and offspring mice were fed for a total of 5 weeks after birth. All groups were fed with the diet and water with free access. The overall environmental conditions were as follows: natural illumination for 12 h per day, a temperature of (21.94 ± 0.31) °C, and humidity of (53.00 ± 1.67)%. Acclimatization of offspring mice was consistent with the maternal mice.

Once maternal mice appeared to have a copulation plug after mating with male mice, which were housed individually, they were considered pregnant. The size of the cage we used was as follows: the cage is small (AJY Life Sciences Co., Ltd., Nanjing, China), with clasps to prevent the mice from escaping, and there is a place where we can put the water bottle to provide water to the mice. The whole cage body is made of polypropylene (PP), the cage cover is 304 stainless steel, and the overall size is 32 cm × 20.2 cm × 13.5 cm. This size of cage can accommodate up to 5–8 mice. During gestation and lactation, the maternal mice maintained the diet corresponding to each group. After the maternal mice delivered offspring, the litter lived with the maternal mice until the end of lactation. After 5 weeks following birth, the maternal and offspring mice were sacrificed by anaesthetisation with isoflurane (RWD Scientific Co., Ltd., Nanjing, China).

### 2.2. Growth and Reproductive Performance Measurement and Samples Collection

At the beginning of the study, body weights of all the maternal mice were measured. Subsequently, weekly weight and daily food intake were measured. Measurement was the same for offspring mice. When the maternal mice delivered, the litter size was recorded. The weaned offspring mice lived with the maternal mice until they were 3 weeks old. During dissection, body and intestinal length data, body weight, organ weight, abdominal fat weight, intestinal weight, collection of chyme samples from colon for microbial composition, and segment of colon for staining were recorded. After the experiment, the food efficiency ratio (FER), organ index (liver, spleen, kidney, and abdominal fat), intestinal length and weight to body weight ratio, and survival rate were calculated.

### 2.3. Intestinal Histology

Tissues of the colon and liver for haematoxylin and eosin (HE) staining were fixed in 4% solution of paraformaldehyde (Biosharp Life Sciences Co., Ltd., Hefei, China). The process lasted for 24 h. Colon tissues for periodic acid–Schiff (PAS) staining were fixed in Carnoy’s fixative (Wabcan Scientific Co., Ltd., Fuzhou, China), and tissues were stained using HE and PAS staining. Images were acquired by light microscopy using a NIKON ECLIPSE E100 and photographed at magnifications of 200× and 100×. The villus length and crypt depth were measured using ImageJ software (version 2.1.0\1.53c) and the villus length to crypt depth ratio (V/C) was subsequently calculated.

### 2.4. 16S rRNA Sequencing

The chyme samples from the colon were collected and stored in an ultra-low-temperature freezer with the temperature set to −80 °C. The number of biological repeats was 5 in every group, and DNA was extracted by the CTAB/SDS method. The V3–V4 region of the 16S rRNA was amplified with the universal primers 341F and 806R. Sequencing libraries were constructed, qualified, and sequenced using the NEBNext^®^ Ultra™ IIDNA Library Prep Kit (Cat No. E7645) and Qubit@ 2.0 Fluorometer (Thermo Scientific, Shanghai, China). FLASH (V1.2.11) was used to merge reads with overlapping regions and the splicing sequences, then, using fastp (Version 0.20.0), OTUs with more than 97% similarity were clustered. A representative sequence for each OTU was screened for further annotation. A Naive Bayes classifier algorithm was used to annotate taxonomic information for each sequence.

### 2.5. Statistical Analyses

The data were analysed using SPSS Statistics software (version 21.0.0) to compare the significance among the three groups using one-way analysis of variance, followed by Duncan’s multiple comparison test. Data are presented as mean ± SEM, with *p* < 0.05 considered statistically significant.

## 3. Results

### 3.1. Effects of Pea Fibre on Growth and Development in Maternal and Offspring Mice

**Figure 2:**
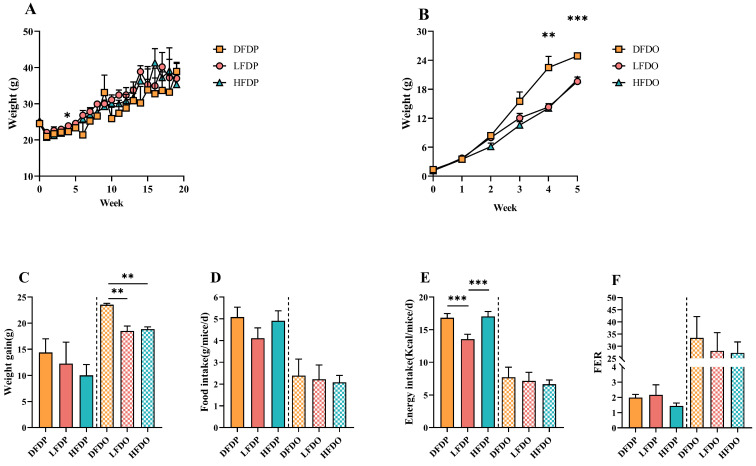
Effects of pea fibre on growth and development of maternal and offspring mice. (**A**) Body weight of maternal mice; (**B**) body weight of offspring mice; (**C**) weight gain; (**D**) food intake; (**E**) energy intake; (**F**) food efficiency ratio (FER) = [total body weight gain (g)]/[total food intake (g)] × 100. * *p* < 0.05, ** *p* < 0.01, *** *p* < 0.001. DFDP: deprived-fibre-diet parent mice; LFDP: low-fibre-diet parent mice; HFDP: high-fibre-diet parent mice; DFDO: deprived-fibre-diet offspring mice; LFDO: low-fibre-diet offspring mice; HFDO: high-fibre-diet offspring mice.

The effects of pea fibre on the growth performance of maternal and offspring mice are shown in Figure 2. The initial body weights of the maternal mice were similar among the groups. During the 20 weeks of feeding, maternal mice in the three groups showed different increases in body weight (Figure 2A). Weight gain was higher in the DFDP group than in the HFDP and LFDP groups (Figure 2C). The food intake of the DFDP group was higher than the intake of the HFDP and LFDP groups (Figure 2D). Additionally, compared to the LFDP groups, energy intake in the DFDP and HFDP groups was significantly high (*p* < 0.001) (Figure 2E), whereas the food efficiency ratio (FER) in the LFDP group was higher than the FER in the DFDP and HFDP groups (Figure 2F).

Compared to the maternal group, the indicators of growth performance among the offspring improved. During the period of weaning (4–5 weeks of age), the body weight of the DFDO group was significantly higher than the LFDO and HFDO groups (4 weeks of age, *p* < 0.01; 5 weeks of age, *p* < 0.001) (Figure 2B). There were significant differences in weight gain (*p* < 0.01) among the DFDO, LFDO, and HFDO groups (Figure 2C). There were no significant differences of food intake and energy intake among offspring (Figure 2D,E). The FER in the DFDO group was higher than the LFDO and HFDO groups (Figure 2F). Our research indicates that DF deprivation in parents may induce obesity in offspring.

### 3.2. Effects of Pea Fibre on Maternal Reproductive Performance

**Figure 3:**
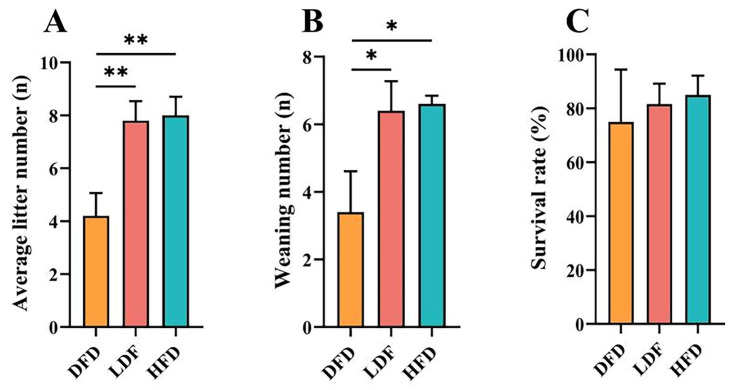
Effects of pea fibre on maternal reproductive performance parental mice. (**A**) average litter number; (**B**) weaning number; (**C**) survival rate. * *p* < 0.05, ** *p* < 0.01. DFD: deprived fibre diet; LFD: low fibre diet; HFD: high fibre diet. survival rate = [The number of weaning offspring]/[the total number of offspring born] × 100.

The effects of pea fibre on maternal reproductive performance are shown in Figure 3. Compared with the DFD group, offspring in the LFD and HFD groups had significantly higher average litter numbers at birth (*p* < 0.01) (Figure 3A) and weaning numbers at 21 days of age (*p* < 0.05) (Figure 3B). There was no statistical difference in survival rates among three groups, and the survival rates of the offspring in the LFD and HFD groups were higher than the DFD group (Figure 3C). The findings indicate that DF deprivation can decrease the reproductive performance of female mice, especially the average litter size and number of weaned mice.

### 3.3. Effects of Pea Fibre on Organ and Intestinal Indices in Mice

**Figure 4:**
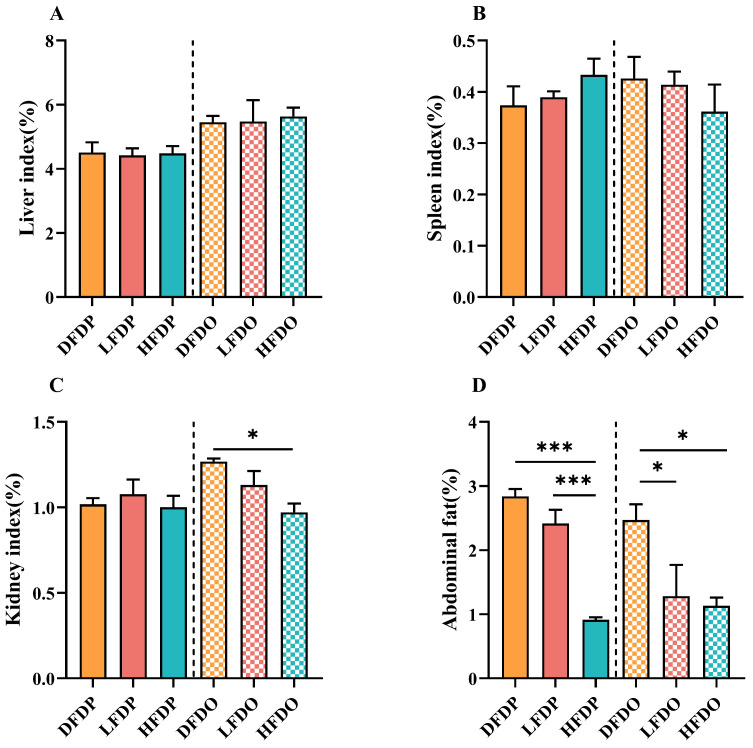
Effects of pea fibre on relative organ indices of parental and offspring mice. (**A**) Liver index; (**B**) spleen index; (**C**) kidney index; (**D**) abdominal fat index. * *p* < 0.05, *** *p* < 0.001. Organ indices = [organ (g)]/[body weight (g)] × 100. DFDP: deprived-fibre-diet parent mice; LFDP: low-fibre-diet parent mice; HFDP: high-fibre-diet parent mice; DFDO: deprived-fibre-diet offspring mice; LFDO: low-fibre-diet offspring mice; HFDO: high-fibre-diet offspring mice.

Relative organ and intestinal indices of the mice are shown in Figure 4. There were no statistical differences in the liver and spleen indices between the parental and offspring mice (Figure 4A,B). The kidney index was significantly lower in the HFDO group than he DFDO group (Figure 4C). In addition, the abdominal tissue of HFDP mice was significantly lower than that of DFDP and LFDP groups (*p* < 0.001) in parental mice. In the offspring mice, the abdominal tissue in the LFDO and HFDO groups were significantly lower than the DFDO group (*p* < 0.05) (Figure 4D). These findings indicate that pea fibre can significantly reduce the amount of abdominal fat in parental and offspring mice.

**Figure 5:**
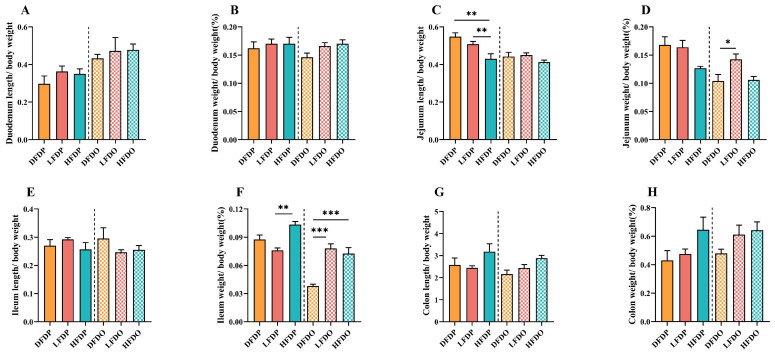
Effects of pea fibre on index of different intestinal segments among maternal and offspring mice. (**A**) Duodenum length to body weight ratio; (**B**) duodenum weight to body weight ratio; (**C**) jejunum length to body weight ratio; (**D**) jejunum weight to body weight ratio; (**E**) ileum length to body weight ratio; (**F**) ileum weight to body weight ratio; (**G**) colon length to body weight ratio; (**H**) colon weight to body weight ratio. * *p* < 0.05, ** *p* < 0.01, *** *p* < 0.001. Intestinal segment length to body weight ratio= [intestinal segment length (cm)]/[body weight (g)] × 100; intestinal segment weight to body weight ratio= [intestinal segment (g)]/[body weight (g)] × 100. DFDP: deprived-fibre-diet parent mice; LFDP: low-fibre-diet parent mice; HFDP: high-fibre-diet parent mice; DFDO: deprived-fibre-diet offspring mice; LFDO: low-fibre-diet offspring mice; HFDO: high-fibre-diet offspring mice.

The effect of pea fibre on intestinal length and weight in maternal and offspring mice is depicted in Figure 5. There was no significant difference in the lengths of the duodenum, ileum, or colon between maternal and offspring mice (*p* > 0.05) (Figure 5A,E,G). The jejunum of maternal mice in the HFDP group was significantly shorter than the length in maternal mice in DFDP and LFDP groups (*p* < 0.05). In the offspring, jejunum length was not significantly different between the groups (Figure 5C). The weights of the duodenum and colon were not significantly different between the maternal and offspring groups (*p* > 0.05) (Figure 5B,H). Jejunum weight in LFDO mice was significantly higher than that in DFDO mice (*p* < 0.05) (Figure 5D). The ileum weights of HFDP were significantly higher than LFDP mice (*p* < 0.01), and ileum weights of HFDO and LFDO mice were significantly higher than that of DFDO mice (*p* < 0.001) (Figure 5F). Addition of pea fibre had little effect on the weight and length of intestinal segments per unit body weight.

### 3.4. Effects of Pea Fibre on Histopathology in Maternal and Offspring Mice

**Figure 6:**
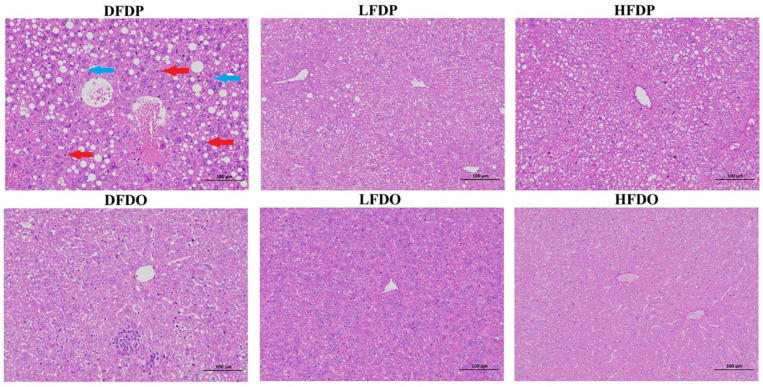
Effects of pea fibre on liver histopathological of maternal and offspring mice. Representative micrographs of HE staining of liver (original magnification 200×, scale bar 100 μm). Blue arrow: lipid droplet vacuole; Red arrows: lymphocyte. DFDP: deprived-fibre-diet parent mice; LFDP: low-fibre-diet parent mice; HFDP: high-fibre-diet parent mice; DFDO: deprived-fibre-diet offspring mice; LFDO: low-fibre-diet offspring mice; HFDO: high-fibre-diet offspring mice.

The effects of pea fibre on liver histopathology are presented in Figure 6. Compared to LFDP, DFDP hepatocytes contained a large number of lipid droplet vacuoles (blue arrows) and increased lymphocyte numbers (red arrows). In contrast, HFDP hepatocytes showed improved hepatic cords, a small amount of cell degeneration, and occasional lipid droplet vacuoles. Compared with the maternal groups, the structure of the hepatic lobules was relatively clear and the hepatocyte lipid droplet vacuoles and inflammatory cells of the offspring mice were significantly reduced, while the hepatocytes of DFDO still showed obvious inflammatory cell and lymphocyte infiltration compared with the LFDO and HFDO groups. In the LFDO and HFDO groups, the cell cytoplasm was arranged in an orderly manner. However, liver injury was apparent in the DFDO group, as evidenced by infiltration, cell balloon-like appearance of hepatocytes, and hepatocellular necrosis. The findings indicate that DF deprivation caused fatty-liver-like lesions and inflammatory infiltration in maternal and offspring mice.

**Figure 7:**
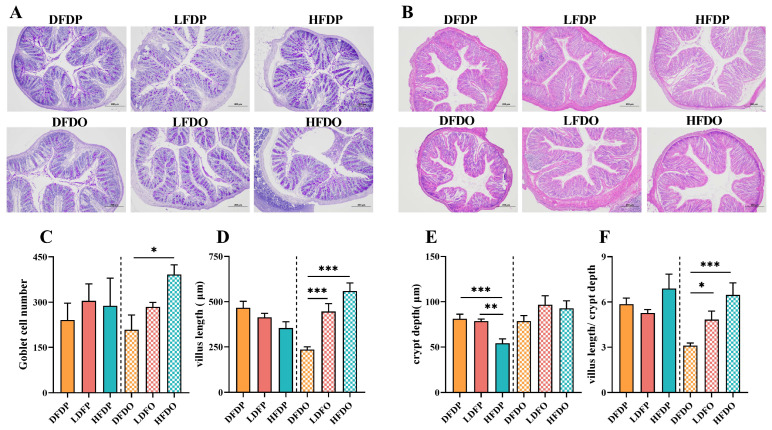
Effects of pea fibre on colon morphology within colons of maternal and offspring mice. (**A**) PAS staining of mice colon (original magnification 100×, scale bar 200 μm); (**B**) HE staining of mice colon (original magnification 100× (scale bar 200 μm); (**C**) number of goblet cells in maternal and offspring mice; (**D**) villus length of maternal and offspring mice; (**E**) crypt depth ratio of maternal and offspring mice; (**F**) villus length to crypt depth ratio (V/C) of maternal and offspring mice. * *p* < 0.05, ** *p* < 0.01, *** *p* < 0.001. DFDP: deprived-fibre-diet parent mice; LFDP: low-fibre-diet parent mice; HFDP: high-fibre-diet parent mice; DFDO: deprived-fibre-diet offspring mice; LFDO: low-fibre-diet offspring mice; HFDO: high-fibre-diet offspring mice.

Morphology data of the colonic tissue are presented in Figure 7. Goblet cells in the colon in the DFDP group were no different compared to the number in the HFDP group. Moreover, among the offspring, there were significantly more goblet cells in mice in the HFDO group than the DFDO group (*p* < 0.05) (Figure 7C). The villus length in the HFDO, LFDO group was significantly longer than the villus length in mice in the DFDO group (*p* < 0.001) (Figure 7D). The crypt depth in the HFDP group was significantly lower than that in the DFDP group (*p* < 0.001) and LFDO group (*p* < 0.01) (Figure 7E). the V/C in the HFDO group was significantly greater than that in the DFDO group (*p* < 0.001) and LFDO group (*p* < 0.05) (Figure 7F). The findings demonstrate that DF deprivation reduced goblet cells and their secreted mucus barrier in the intestines of maternal and offspring mice, and led to more pronounced intestinal villi structural dysplasia in the offspring.

### 3.5. Effects of Pea Fibre on Differences in Microbiota Composition in Maternal and Offspring Mice

**Figure 8:**
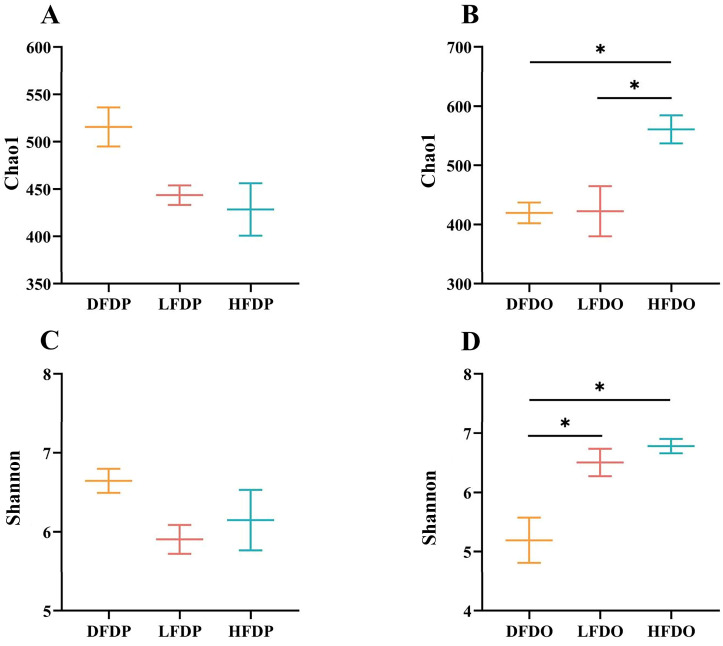
Effects of pea fibre on α-diversity of gut microbiota. (**A**) Chao1 index in maternal mice; (**B**) Chao1 index in offspring mice; (**C**) Shannon index in maternal mice; (**D**) Shannon index in offspring mice. * *p* < 0.05. DFDP: deprived-fibre-diet parent mice; LFDP: low-fibre-diet parent mice; HFDP: high-fibre-diet parent mice; DFDO: deprived-fibre-diet offspring mice; LFDO: low-fibre-diet offspring mice; HFDO: high-fibre-diet offspring mice.

The α-diversity measured by the indicator revealed no differences of species diversity among the groups for the maternal mice. The Chao1 index had a lower trend in LFDP and HFDP groups than DFDP group (Figure 8A). The Shannon index had a higher trend in DFDP group compared LFDP and HFDP groups (Figure 8C). In contrast, the Chao1 indices indicated that the microbiome diversity of mice in the HFDO group was significantly greater than that the DFDO, LFDO group (*p* < 0.05) (Figure 8B). The Shannon index of the HFDO and LFDO groups were significantly higher than the DFDO value (*p* < 0.05) (Figure 8D). The findings indicate that the diversity of gut microbiota in parental mice decreased after DF deprivation, while the diversity of microbiota in offspring further decreased. Supplementation with pea fibre increased the diversity of gut microbiota in parental and offspring mice, and the gut microbiota diversity of offspring was significantly higher among high-fibre-diet offspring mice.

**Figure 9:**
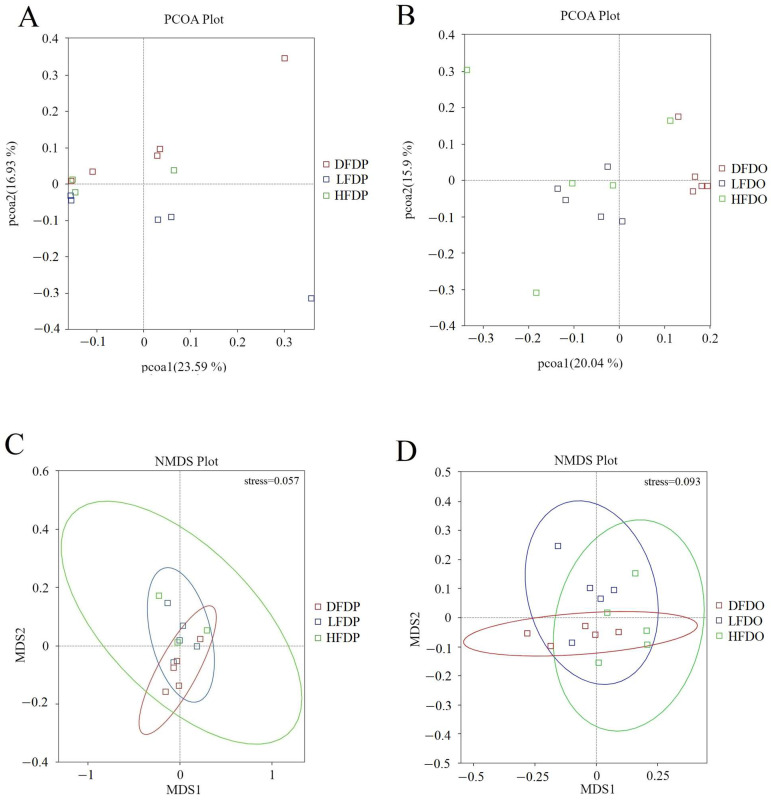
Effects of pea fibre on β-diversity of gut microbiota. (**A**) PCoA plot based on unweighted UniFrac in maternal mice; (**B**) PCoA plot based on unweighted UniFrac in offspring mice; (**C**) NMDS plot based on weighted UniFrac in maternal mice; (**D**) NMDS plot based on weighted UniFrac in offspring mice. DFDP: deprived-fibre-diet parent mice; LFDP: low-fibre-diet parent mice; HFDP: high-fibre-diet parent mice; DFDO: deprived-fibre-diet offspring mice; LFDO: low-fibre-diet offspring mice; HFDO: high-fibre-diet offspring mice.

ANOSIM analysis revealed significant differences in the operational taxonomic units in samples from the DFDP and LFDP groups (*R* = 0.19, *p* = 0.03) (Figure 9A,C). Significant differences were observed among the offspring in the HFDO and DFDO groups (*R* = 0.33, *p* = 0.01) and the differences between LFDO and DFDO groups (*R* = 0.52, *p* = 0.01) (Figure 9B,D).

**Figure 10:**
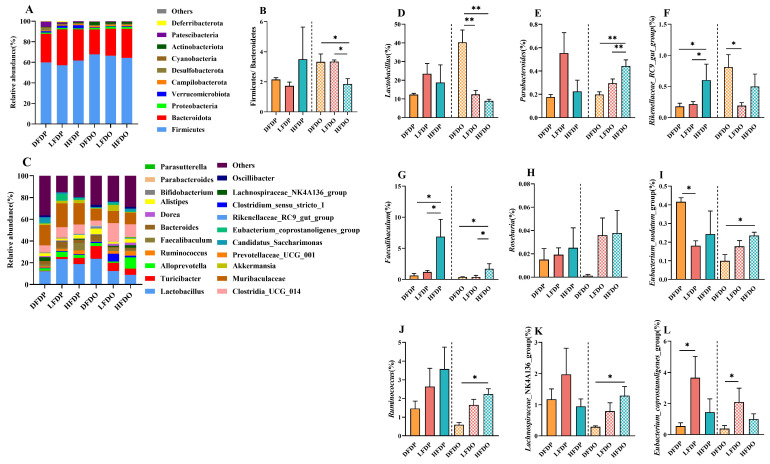
Effects of pea fibre on relative abundance of bacteria. (**A**) colonic microflora structure at phylum level; (**B**) ratio of Firmicutes to Bacteroidetes; (**C**) colonic microflora structure at genus level; (**D**–**L**) significant differential bacteria at genus level in mice between generations of each group. * *p* < 0.05, ** *p* < 0.01. DFDP: deprived-fibre-diet parent mice; LFDP: low-fibre-diet parent mice; HFDP: high-fibre-diet parent mice; DFDO: deprived-fibre-diet offspring mice; LFDO: low-fibre-diet offspring mice; HFDO: high-fibre-diet offspring mice.

The effect of pea fibre on the abundance of colonic microflora is shown in Figure 10. The dominant phyla in all groups were Firmicutes, Bacteroidetes, Proteobacteria, and Verrucomicrobiota (Figure 10A). Firmicutes/Bacteroidetes of the HFDP group were higher than the DFDP and LFDP groups. However, Firmicutes/Bacteroidetes of the HFDO group was significantly lower than the DFDO and LFDO groups (*p* < 0.05) (Figure 10B). At the genus level, *Lactobacillus*, *Bacteroides*, and *Clostridia_UCG*_*014* were the most prevalent genera in every group (Figure 10C), with *Lactobacillus* being more prevalent in the LFDP and HFDP groups than in the DFDP group. However, in the offspring group, the prevalence of *Lactobacillus* in the DFDO group was significantly higher than LFDO and HFDO groups (*p* < 0.01) (Figure 10D). Abundance of *Parasutterella* showed the same trend in the maternal group (Figure 10C). By comparing the different species of intestinal microflora among the groups, the changes in the intestinal microflora were analysed at the genus level. *Faecalibaculum*, *Roseburia*, and *Ruminococcus* in the HFDP and HFDO groups were higher than in other maternal and offspring groups (Figure 10G,H,J). *Faecalibaculum* in the HFDP group was significantly higher than in DFDP and LFDP groups (*p* < 0.05). *Faecalibaculum* and *Ruminococcus* in the HFDO group were significantly higher than in the DFDO group (*p* < 0.05). *Parabacteroides* and *Lachnospiraceae_NK4A136_group* in maternal mice were not significantly different (Figure 10E,K). Among the offspring group, *Parabacteroides* in the HFDO group was significantly more abundant than in the DFDO and LFDO groups (*p* < 0.01) (Figure 10E), and *Lachnospiraceae_NK4A136_group* was significantly higher in the HFDO group compared to the DFDO group (*p* < 0.05) (Figure 10K). *Lactobacillus* in the DFDO group was significantly more abundant than in the LFDO and HFDO groups (*p* < 0.01) (Figure 10D), and *Rikenellaceae_RC9_gut_group* was more prevalent in maternal mice in the HFDP group than in the DFDP and LFDP groups (*p* < 0.05). However, in offspring mice, *Rikenellaceae_RC9_gut_group* was more abundant in the DFDO group than the LFDO group (Figure 10F). The *Eubacterium_coprostanoligenes_group* was significantly more abundant in the LFDP and LFDO groups compared to the DFDP and DFDO groups, respectively (*p* < 0.05) (Figure 10L). Finally, the *Eubacterium_nodatum_group* was more abundant in the DFDP group than in the LFDP group (*p* < 0.05), and in the HFDO group more than in the DFDO group (*p* < 0.05) (Figure 10I).

**Figure 11:**
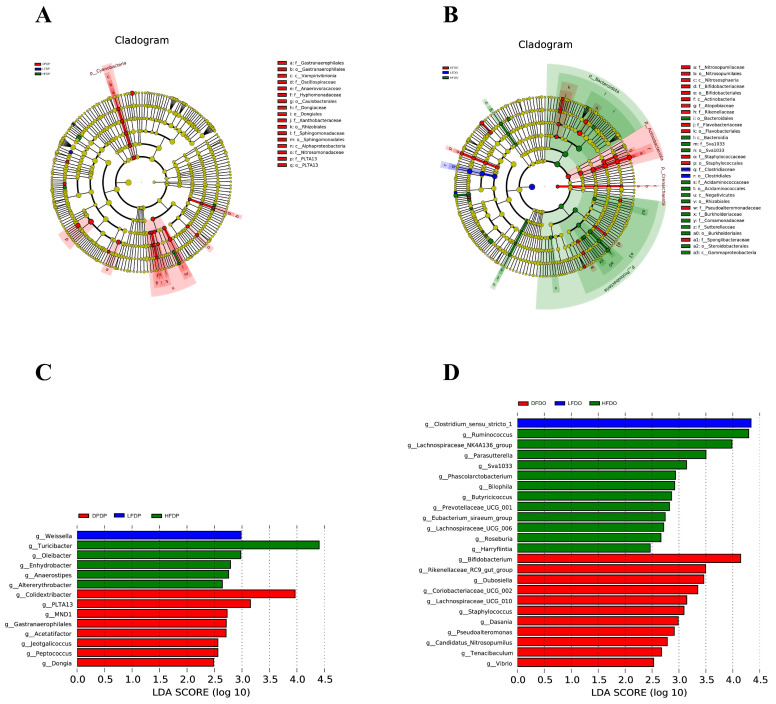
Effects of pea fibre on LEfSe and LDA analysis. (**A**) Evolutionary branching diagram in maternal mice; (**B**) evolutionary branching diagram in offspring mice; (**C**) LDA analysis in maternal mice with LDA > 2; (**D**) LDA analysis in offspring mice with LDA > 2. DFDP: deprived-fibre-diet parent mice; LFDP: low-fibre-diet parent mice; HFDP: high-fibre-diet parent mice; DFDO: deprived-fibre-diet offspring mice; LFDO: low-fibre-diet offspring mice; HFDO: high-fibre-diet offspring mice.

Linear discriminant analysis effect size (LEfSe) and linear discriminant analysis at the genus level showed the enrichment of *Weissella* in HFDP mice, *Turicibacter* in HFDP mice, and *Colidextribacter* in DFDP mice (Figure 11A,C). Among offspring, some species of fibre-degrading microflora were enhanced in mice fed pea fibre; *Clostridium_sensu_stricto_1* was enriched in LFDO mice, *Ruminococcus* in HFDO mice, and *Bifidobacterium* in DFDO mice (Figure 11B,D).

## 4. Discussion

Peas are a legume that are preferred by consumers because of their nutritional ingredients that include protein, starch, fibre, minerals, and vitamins. These ingredients are considered beneficial to intestinal health owing to their nutritional characteristics, such as improved glycaemic index and plasma lipids [16,17]. DF is an important nutrient that regulates intestinal health. Different types of DF have potential impacts on individual indicators, such as body weight [18,19]. In this study, pea fibre influenced offspring body weight, especially during weaning. The body weight of fibre-deprived mice was significantly higher than that of high-fibre-fed mice. Our research findings also support the finding that DF deprivation can lead to obesity in offspring. Supplemental DF affected individual body weight and longevity. Yu et al. [20] found that fibre deprivation can significantly increase the body weight of naturally aged mice. In our study, the same trend was observed in fibre-deprived mice. In addition, increased abdominal fat and injured liver tissue seem to illustrate the effect of fibre deprivation on obesity. We also observed that in mice fed a diet supplemented with pea fibre, especially offspring mice, in addition to the changes in weight, the accumulation of abdominal fat in the body and lipids in the liver were significantly reduced. These findings reveal that pea fibre can prevent and alleviate obesity. Therefore, pea fibre intake may be conducive to longevity and fibre deprivation may affect individual longevity during the late stages of growth. In a recent study involving a mouse model of non-alcoholic fatty liver disease (NAFLD), the diet was supplemented with pea hulls. The results of multi-omics analyses showed NAFLD was improved by the serine hydroxy methyltransferase 2/glycine/mammalian target of rapamycin/peroxisome proliferator-activated receptor gamma (SHMT2/glycine/mTOR/PPAR-γ) signalling pathway, with the increasing contents of glutathione, total antioxidant capacity, and adiponectin, which may improve oxidative stress, inhibit lipid peroxidation, and reduce the risk of obesity metabolic abnormalities. In addition, the level of total glyceride, total cholesterol, interleukin-lβ (IL-1β), and IL-6 decreased in liver tissue, which may improve the insulin-related reaction [21]. The findings reveal that pea fibre may prevent obesity through the SHMT2/glycine/mTOR/PPAR-γ signal pathway. The mechanism remains unresolved.

With the emergence of the concepts of “mother-offspring-integration” and “One Health”, researchers have become concerned about mother–offspring nutrition. This concern has spurred the recognition of the importance of fibre, which can effectively improve host and offspring gut microorganisms [22]. In practice, maternal nutrition during pregnancy affects the growth and health of the offspring, which is specifically manifest in growth, development, and reproduction of the offspring. Imbalanced nutrition in the parental generation can induce some diseases in offspring [23,24]. In our study, the addition of pea fibre to the diet significantly increased the average litter number at birth and weaning number at 21 days of age. DF has been shown to have a significant influence on reproductive performance. However, the effects of DF on reproductive performance in mice are unclear. Li et al. [25] found that sows fed diets containing inulin and cellulose displayed significantly influenced reproductive performance during three successive cycles; the average weight of pigs and litters during the birth and weaning phases was significantly higher than control group. Furthermore, Loisel et al. [26] found the mortality of offspring before weaning was significantly increased in the gestation group fed feed containing 23.4% total DF compared with the low-fibre group (13.3% total DF). Deprivation of DF and high DF can reduce reproductive performance in mice. Further research is needed to determine the amount of pea fibre required for mice to achieve optimal reproductive performance, and how pea fibre regulates reproductive performance.

Villus length influences the capability to absorb nutrients, which relies on the absorptive area and crypt depth, which in turn indicate the maturation rate of cells. Therefore, villus length/crypt depth is considered to be related to digestion and absorption capacity [27]. We found that pea fibre improved the ratio of villus length to crypt depth, which showed that the villus length increased significantly among offspring mice supplemented with pea fibre, but the crypt depth was higher than that in offspring mice fed a fibre-deprived diet. Maternal mice fed a high-fibre diet displayed the shortest villus length. However, the reason remains unknown. Goblet cells secrete mucus into the intestinal epithelium after injury, which can maintain intestinal integrity [28]. During this period, there was no remarkable difference in goblet cell numbers in the colon. However, after DF deprivation, offspring showed a significant decrease in goblet cells compared to high-fibre-diet offspring. Knapp et al. [29] reported that soluble fibre dextrin and soluble corn fibre can significantly increase the total number of goblet cells per crypt in rats compared to the control group. We speculate that the increased number of cells reflects the acidification of colonic contents and the production of a series of metabolites arising from the synthesis of mucus [30]. The benefits of pea fibre for intestinal health have been studied previously in piglets. In the study, pea fibre significantly increased goblet cells compared to control group, even though the villus length and crypt depth did not differ from the control group [31]. However, pea fibre can improve crypt depth and goblet cells in the colon.

Aside from its role in nutrient digestion and absorption, the intestine is also an important immune organ for humans and animals. The intestine is considered the second brain of the body, with reports of the bidirectional communication between the intestinal microflora and the brain via the gut–brain axis [32,33]. Microbial communities colonise the microflora as a function of self-regulation through mutual interaction [34]. The intestinal microflora are important in maintaining host health, enhancing the body’s immunity, providing conducive metabolites to the host, and resisting colonisation by pathogenic bacteria [35,36,37]. In the present study, relative abundance of the colonic microflora in all groups differed. At the phylum level, the Firmicutes/Bacteroidetes ratio in the maternal mice showed no significant differences among the groups. However, among offspring, this ratio was significantly lower in the HFDO group than DFDO and LFDO groups. This trend was also reported in a study on the effects of peas on the rat microflora. The authors described that raw or cooked pea fibre fed to rats increased the relative abundance of Firmicutes and decreased the relative abundance of Bacteroidetes compared to the group fed a high-fat diet [38]. As the key microflora in the host intestine, Bacteroides and Firmicutes are related to the energy acquisition process. Thus, changes in these microorganisms will affect the host’s glucose homeostasis, anti-inflammatory activity, and other reactions.

DF can help optimise the structure and increase the diversity of the gut microbiota. A diverse gut microbiota can resist infection by exogenous pathogenic microorganisms, promoting intestinal health [3,22,37]. Our research also confirmed that supplementation with pea fibre can increase the diversity of the gut microbiota in both parental and offspring mice. Other studies have reported that DF can improve the host gut microflora, and most microflora can produce SCFAs. These SCFAs include acetate, butyrate, and lactate, among others, which are profoundly important in the gut; the fluctuating content of SCFAs reflects their production by the particular bacteria [39,40]. In the present study, at the genus level, significant changes in *Lactobacillus*, *Parabacteroides*, and *Rikenellaceae_RC9_gut*_group were observed in offspring mice. With increasing focus the relationship between intestinal microflora and neuropsychiatric diseases, researchers have found that intestinal microflora can regulate the development and behaviour of the brain through the brain–gut–microbiota axis, which influences some diseases, such as autism, spectrum disorders, depression, Parkinson’s disease, and other psychiatric diseases [41,42]. We found that under the influence of maternal potential, the genera *Parabacteroides*, *Akkermansia*, and *Prevotellaceae*_ *UCG_ 001* decreased, and *Lactobacillus*, *Alistipes* increased after DF deprivation, with the same tendencies evident in the offspring. The findings are similar to those of previous studies on neuropsychiatric diseases [43,44,45]. Furthermore, the early period of growth in autism may be related to early feeding methods; infants who were weaned early showed increased concentrations of propionate and butyrate in faeces, and changes in SCFAs may induce neuropsychiatric diseases [46]. Butyrate can activate G-protein-coupled receptors and can affect neuropsychiatric diseases [47]. The tendency for DF deprivation in offspring mice may lead to psychiatric diseases later in life. Studies on inflammatory bowel diseases, including Crohn’s disease and ulcerative colitis, have found that the microflora may affect the occurrence of these diseases through the microbiota-gut-brain axis [48,49]. We found that some SCFA-producing bacteria were lower under DF deprivation in maternal and offspring mice. The observation that *Roseburia* and *Ruminococcus* were lower than in the other groups indicates that pea fibre deprivation reduced SCFAs production in mice. Li et al. [50] found a decreased abundance of *Roseburia* and *Ruminococcus* in patients with ulcerative colitis. Li et al. [25] reported that Lachnospiraceae was negatively correlated with inflammation status. Available evidence suggests that disordered microflora is related to homeostasis in the gut. We observed no significant difference in *Lachnospiraceae_NK4A136_ group* among maternal mice. However, a significant difference was evident in offspring mice fed a diet including high pea fibre compared to dietary-fibre-deprived offspring mice. These findings indicate that deprivation of fibre changed the intestinal microflora. The characteristic change was similar to that of intestinal microflora in patients with neuropsychiatric diseases and inflammatory bowel diseases. However, more research is needed for clarification. Pea fibre can improve the composition of the intestinal microflora, with increased contents of SCFA-producing bacteria and cellulose-degrading bacteria. Additionally, SCFAs can regulate the central nervous system via gastrointestinal hormones, such as leptin and peptide YY, and influence neuropsychiatric and inflammatory diseases through interactions with free fatty acid receptors [51]. Therefore, nutrients, microflora, and hormones can affect homeostasis.

A recent study indicated that offspring reflect the maternal microbiota via vertical transmission during the perinatal period [52]. In our study, the gut microbiota among maternal mice was transferred during pregnancy to their offspring. The latter mice in the HDFO and DFDO groups displayed altered gut microbiota. This tendency was similar with the maternal groups, which may have resulted from the consumption of pea fibre. We also observed that dysbacteriosis delivered to offspring of fibre-deprived mice profoundly contributed to neuropsychiatric diseases, consistent with previous studies [53]. Lin et al. [54] reported that supplying tributyrin in late pregnancy and during lactation can improve the faecal microbiota and relative abundance of *Lactobacillus* in sows and offspring, and that the *Eubacterium_fissicatena_group* decreased among piglets in the tributyrin group. Maternal gut microbiota can have more lasting effects on the gut microbiota than other sources [2].

## 5. Conclusions

Pea fibre deprivation in mice can inhibit intestinal development; decrease the reproductive performance of maternal mice; decrease the relative abundance of *Lachnospiraceae_NK4A136_group*, *Roseburia*, *Ruminococcus*, and *Parabacteroides*; and increase the relative abundance of *Lactobacillus* and *Rikenellaceae_RC9_gut_group* in the colon of offspring mice. Deprivation of DF in maternal mice also increases the risk of obesity and other intestinal diseases in their offspring. Supplementation with pea fibre can alleviate the possibility of obesity in mice fed with high pea fibre in maternal and offspring mice, as well as improving intestinal segments, histopathology, release of fat deposition, and potential intestinal microflora associated with diseases. The collective findings indicate the important roles of pea fibre in preventing obesity, improving reproductive performance, and reshaping the gut microbiota in mice.

## Figures and Tables

**Table 1:** Ingredients and nutritional level of the experimental diets (as fed basis).

Item	Experimental Diets ^1^		
	DFD	LFD	HFD
Ingredients, %			
Maize starch	50.55	50.75	43.25
Fish meal	13.22	13.22	13.22
Wheat flour	5.00	5.00	5.00
Lard	5.20	5.00	7.50
DL-Methionine	0.20	0.20	0.20
Limestone	0.10	0.10	0.10
Dicalcium phosphate	0.10	0.10	0.10
Sodium chloride	0.13	0.13	0.13
Choline chloride	0.20	0.20	0.20
Casein	10.00	10.00	10.00
Sucrose	10.00	10.00	10.00
Pea fibre ^2^	0.00	5.00	10.00
Vitamin and mineral premix ^3^	0.30	0.30	0.30
Bentonite	5.00	0.00	0.00
Calculated nutrient level			
Metabolic energy, MJ/kg	13.53	13.48	13.40
Crude protein, %	18.04	18.04	18.02
Dietary fibre, %	0.08	5.03	9.98
Lysine, %	1.51	1.51	1.49
Methionine, %	0.76	0.76	0.75
Calcium, %	0.60	0.60	0.60
Available phosphorus, %	0.43	0.43	0.43

^1^ DFD: deprived fibre diet; LFD: low fibre diet; HFD: high fibre diet. ^2^ Pea fibre contains 96.07% total dietary fibre, 50.93% soluble dietary fibre, 45.14% insoluble dietary fibre. ^3^ Per kg of the diet provided as following: vitamin A (retinol) 2880 IU, vitamin B_1_ 6 mg, vitamin B_2_ 8.4 mg, vitamin B_12_ (cyanocobalamin) 0.01 mg, vitamin D_3_ (cholecalciferol) 1440 IU, vitamin E (dl-alpha tocopherol acetate) 24 mg, vitamin K_3_ (Menadione) 1.2 mg, vitamin B_7_ 0.24 mg, vitamin B_9_ 0.6 mg, vitamin B_5_ (D-calcium pantothenate) 19.20 mg, vitamin B_3_ 18 mg, vitamin B_6_ 9.6 mg, Cu (as CuSO_4_·5H_2_O) 6 mg, Fe (as FeSO_4_·7H_2_O) 35 mg, Mn (as MnSO_4_·H_2_O) 10 mg, Zn (as ZnSO_4_·7H_2_O) 10 mg, I (as KI) 0.15 mg, Se (as Na_2_SeO_4_) 0.15 mg.

## Data Availability

The data presented in this study will be available upon request from the corresponding author.

## References

[1] Makki K., Deehan E.C., Walter J., Bäckhed F. (2018). The impact of dietary fiber on gut microbiota in host health and disease. Cell Host Microbe.

[2] Cao J., Wang K., Li N., Zhang L., Qin L., He Y., Wang J., Qu C., Miao J. (2023). Soluble dietary fiber and cellulose from *Saccharina japonica* by-product ameliorate Loperamide-induced constipation via modulating enteric neurotransmitters, short-chain fatty acids and gut microbiota. Int. J. Biol. Macromol..

[3] Desai M.S., Seekatz A.M., Koropatkin N.M., Kamada N., Hickey C.A., Wolter M., Pudlo N.A., Kitamoto S., Terrapon N., Muller A. (2016). A dietary fiber-deprived gut microbiota degrades the colonic mucus barrier and enhances pathogen susceptibility. Cell.

[4] Milani C., Duranti S., Bottacini F., Casey E., Turroni F., Mahony J., Belzer C., Delgado Palacio S., Arboleya Montes S., Mancabelli L. (2017). The first microbial colonizers of the human gut: Composition, activities, and health implications of the infant gut microbiota. Microbiol. Mol. Biol. Rev..

[5] Astbury S., Song A., Zhou M., Nielsen B., Hoedl A., Willing B.P., Symonds M.E., Bell R.C. (2018). High fructose intake during pregnancy in rats influences the maternal microbiome and gut development in the offspring. Front. Genet..

[6] Dreisbach C., Prescott S., Alhusen J. (2020). Influence of maternal prepregnancy obesity and excessive gestational weight gain on maternal and child gastrointestinal microbiome composition: A Systematic Review. Biol. Res. Nurs..

[7] Rudra S.G., Hanan E., Sagar V.R., Bhardwaj R., Basu S., Sharma V. (2020). Manufacturing of mayonnaise with pea pod powder as a functional ingredient. J. Food Meas. Charact..

[8] Petropoulou K., Salt L.J., Edwards C.H., Warren F.J., Garcia-Perez I., Chambers E.S., Alshaalan R., Khatib M., Perez-Moral N., Cross K.L. (2020). A natural mutation in *Pisum sativum* L. (pea) alters starch assembly and improves glucose homeostasis in humans. Nat. Food.

[9] Liu N., Song Z., Jin W., Yang Y., Sun S., Zhang Y., Zhang S., Liu S., Ren F., Wang P. (2022). Pea albumin extracted from pea (*Pisum sativum* L.) seed protects mice from high fat diet-induced obesity by modulating lipid metabolism and gut microbiota. J. Funct. Foods.

[10] Hansen I., Knudsen K.E.B., Eggum B.O. (1992). Gastrointestinal implications in the rat of wheat bran, oat bran and pea fibre. Br. J. Nutr..

[11] Marinangeli C.P.F., Krause D., Harding S.V., Rideout T.C., Zhu F., Jones P.J.H. (2011). Whole and fractionated yellow pea flours modulate insulin, glucose, oxygen consumption, and the caecal microbiome in Golden Syrian hamsters. Appl. Physiol. Nutr. Metab..

[12] Patnode M.L., Beller Z.W., Han N.D., Cheng J., Peters S.L., Terrapon N., Henrissat B., Le Gall S., Saulnier L., Hayashi D.K. (2019). Interspecies Competition impacts targeted manipulation of human gut bacteria by fiber-derived glycans. Cell.

[13] Yusuf K., Saha S., Umar S. (2022). Health benefits of dietary fiber for the management of inflammatory bowel disease. Biomedicines.

[14] Nasir G., Zaidi S., Tabassum N., Asfaq (2024). A review on nutritional composition, health benefits and potential applications of by-products from pea processing. Biomass Convers. Biorefin..

[15] National Research Counci Subcommittee on Laboratory Animal Nutritionl (1995). Nutrient Requirements of Laboratory Animals.

[16] Dahl W.J., Foster L.M., Tyler R.T. (2012). Review of the health benefits of peas (*Pisum sativum* L.). Br. J. Nutr..

[17] Lambert J.E., Parnell J.A., Han J., Sturzenegger T., Paul H.A., Vogel H.J., Reimer R.A. (2014). Evaluation of yellow pea fibre supplementation on weight loss and the gut microbiota: A randomized controlled trial. BMC Gastroenterol..

[18] Roager H.M., Vogt J.K., Kristensen M., Hansen L.B.S., Ibrügger S., Mærkedahl R.B., Bahl M.I., Lind M.V., Nielsen R.L., Frøkiær H. (2019). Whole grain-rich diet reduces body weight and systemic low-grade inflammation without inducing major changes of the gut microbiome: A randomised cross-over trial. Gut.

[19] German A.J., Holden S.L., Bissot T., Morris P.J., Biourge V. (2010). A high protein high fibre diet improves weight loss in obese dogs. Vet. J..

[20] Yu X., Liang X., Han K., Shi F., Meng N., Li Q. (2022). Anti-aging effect of dietary fiber compound mediated by guangxi longevity dietary pattern on natural aging mice. Nutrients.

[21] Guo F., Xiong H., Tsao R., Wen X., Liu J., Chen D., Jiang L., Sun Y. (2023). Multi-omics reveals that green pea (*Pisum sativum* L.) hull supplementation ameliorates non-alcoholic fatty liver disease via the SHMT2/glycine/mTOR/PPAR-γ signaling pathway. Food Funct..

[22] Martens E.C. (2016). Fibre for the future. Nature.

[23] Alhasan M.M., Cait A.M., Heimesaat M.M., Blaut M., Klopfleisch R., Wedel A., Conlon T.M., Yildirim A.Ö., Sodemann E.B., Mohn W.W. (2020). Antibiotic use during pregnancy increases offspring asthma severity in a dose-dependent manner. Allergy.

[24] Guo Y., Wang Z., Chen L., Tang L., Wen S., Liu Y., Yuan J. (2018). Diet induced maternal obesity affects offspring gut microbiota and persists into young adulthood. Food Funct..

[25] Li Y., He J., Zhang L., Liu H., Cao M., Lin Y., Xu S., Fang Z., Che L., Feng B. (2021). Effects of dietary fiber supplementation in gestation diets on sow performance, physiology and milk composition for successive three parities. Anim. Feed Sci. Technol..

[26] Loisel F., Farmer C., Ramaekers P., Quesnel H. (2013). Effects of high fiber intake during late pregnancy on sow physiology, colostrum production, and piglet performance1. J. Anim. Sci..

[27] Caspary W.F. (1992). Physiology and pathophysiology of intestinal absorption. Am. J. Clin. Nutr..

[28] Jung K., Saif L.J. (2017). Goblet cell depletion in small intestinal villous and crypt epithelium of conventional nursing and weaned pigs infected with porcine epidemic diarrhea virus. Res. Vet. Sci..

[29] Knapp B.K., Bauer L.L., Swanson K.S., Tappenden K.A., Fahey G.C., De Godoy M.R.C. (2013). Soluble fiber dextrin and soluble corn fiber supplementation modify indices of health in cecum and colon of sprague-dawley rats. Nutrients.

[30] Meslin J.-C., Fontaine N., Andrieux C. (1999). Variation of mucin distribution in the rat intestine, caecum and colon: Effect of the bacterial flora. Comp. Biochem. Physiol. Part. A Mol. Integr. Physiol..

[31] Chen H., Mao X., He J., Yu B., Huang Z., Yu J., Zheng P., Chen D. (2013). Dietary fibre affects intestinal mucosal barrier function and regulates intestinal bacteria in weaning piglets. Br. J. Nutr..

[32] Celi P., Cowieson A.J., Fru-Nji F., Steinert R.E., Kluenter A.M., Verlhac V. (2017). Gastrointestinal functionality in animal nutrition and health: New opportunities for sustainable animal production. Anim. Feed Sci. Technol..

[33] Collins S.M., Surette M., Bercik P. (2012). The interplay between the intestinal microbiota and the brain. Nat. Rev. Microbiol..

[34] Egert M., de Graaf A.A., Smidt H., de Vos W.M., Venema K. (2006). Beyond diversity: Functional microbiomics of the human colon. Trends Microbiol..

[35] Hall A.B., Yassour M., Sauk J., Garner A., Jiang X., Arthur T., Lagoudas G.K., Vatanen T., Fornelos N., Wilson R. (2017). A novel *Ruminococcus gnavus* clade enriched in inflammatory bowel disease patients. Genome Med..

[36] Peterson D.A., Frank D.N., Pace N.R., Gordon J.I. (2008). Metagenomic approaches for defining the pathogenesis of inflammatory bowel diseases. Cell Host Microbe.

[37] Spragge F., Bakkeren E., Jahn M.T., Araujo E.B.N., Pearson C.F., Wang X., Pankhurst L., Cunrath O., Foster K.R. (2023). Microbiome diversity protects against pathogens by nutrient blocking. Science.

[38] Hashemi Z., Fouhse J., Im H.S., Chan C.B., Willing B.P. (2017). Dietary Pea Fiber Supplementation Improves Glycemia and induces changes in the composition of gut microbiota, serum short chain fatty acid profile and expression of mucins in glucose intolerant rats. Nutrients.

[39] Morrison D.J., Preston T. (2016). Formation of short chain fatty acids by the gut microbiota and their impact on human metabolism. Gut Microbes.

[40] Cerdó T., Nieto-Ruíz A., García-Santos J.A., Rodríguez-Pöhnlein A., García-Ricobaraza M., Suárez A., Bermúdez M.G., Campoy C. (2023). Current knowledge about the impact of maternal and infant nutrition on the development of the microbiota–gut–brain axis. Annu. Rev. Nutr..

[41] Rea K., Dinan T.G., Cryan J.F. (2020). Gut Microbiota: A perspective for psychiatrists. Neuropsychobiology.

[42] Dinan T.G., Cryan J.F. (2015). The impact of gut microbiota on brain and behaviour: Implications for psychiatry. Curr. Opin. Clin. Nutr. Metab. Care.

[43] Yu S., Wang L., Jing X., Wang Y., An C. (2023). Features of gut microbiota and short-chain fatty acids in patients with first-episode depression and their relationship with the clinical symptoms. Front. Psychol..

[44] Nishiwaki H., Ito M., Hamaguchi T., Maeda T., Kashihara K., Tsuboi Y., Ueyama J., Yoshida T., Hanada H., Takeuchi I. (2022). Short chain fatty acids-producing and mucin-degrading intestinal bacteria predict the progression of early Parkinson’s disease. npj Park. Dis..

[45] Strati F., Cavalieri D., Albanese D., De Felice C., Donati C., Hayek J., Jousson O., Leoncini S., Renzi D., Calabrò A. (2017). New evidences on the altered gut microbiota in autism spectrum disorders. Microbiome.

[46] Tanoue Y., Oda S. (1989). Weaning time of children with infantile autism. J. Autism Dev. Disord..

[47] Nicholson J.K., Holmes E., Kinross J., Burcelin R., Gibson G., Jia W., Pettersson S. (2012). Host-gut microbiota metabolic interactions. Science.

[48] Ananthakrishnan A.N., Bernstein C.N., Iliopoulos D., Macpherson A., Neurath M.F., Ali R.A.R., Vavricka S.R., Fiocchi C. (2018). Environmental triggers in IBD: A review of progress and evidence. Nat. Rev. Gastroenterol. Hepatol..

[49] Sinagra E., Utzeri E., Morreale G.C., Fabbri C., Pace F., Anderloni A. (2020). Microbiota-gut-brain axis and its affect inflammatory bowel disease: Pathophysiological concepts and insights for clinicians. World J. Clin. Cases.

[50] Li Q., Ding X., Liu K., Marcella C., Liu X., Zhang T., Liu Y., Li P., Xiang L., Cui B. (2020). Fecal microbiota transplantation for ulcerative colitis: The optimum timing and gut microbiota as predictors for long-term clinical outcomes. Clin. Transl. Gastroenterol..

[51] De Vadder F., Kovatcheva-Datchary P., Goncalves D., Vinera J., Zitoun C., Duchampt A., Bäckhed F., Mithieux G. (2014). Microbiota-generated metabolites promote metabolic benefits via gut-brain neural circuits. Cell.

[52] Tochitani S., Tsukahara T., Inoue R. (2024). Perturbed maternal microbiota shapes offspring microbiota during early colonization period in mice. Proc. Jpn. Academy. Ser. B Phys. Biol. Sci..

[53] Schei K., Avershina E., Øien T., Rudi K., Follestad T., Salamati S., Ødegård R.A. (2017). Early gut mycobiota and mother-offspring transfer. Microbiome.

[54] Lin Y., Li D., Ma Z., Che L., Feng B., Fang Z., Xu S., Zhuo Y., Li J., Hua L. (2023). Maternal tributyrin supplementation in late pregnancy and lactation improves offspring immunity, gut microbiota, and diarrhea rate in a sow model. Front. Microbiol..

